# Views on the usability and usefulness of the PeerConnect app among Ontario public safety professionals

**DOI:** 10.1177/20552076251384142

**Published:** 2025-10-01

**Authors:** Gillian Foley, Marcella Cassiano, Rosemary Ricciardelli

**Affiliations:** 1 Department of Psychology, Faculty of Science, 120907 Memorial University of Newfoundland, St John's, Newfoundland, Canada; 2 Department of Criminal Justice, 8665 University of Winnipeg, Winnipeg, Manitoba, Canada; 3 School of Maritime Studies, 98014 Marine Institute of Memorial University of Newfoundland, St John's, Newfoundland, Canada

**Keywords:** Peer support, apps, mobile health technology, public safety personnel, first responders

## Abstract

Public safety professionals (PSPs) (e.g. police officers, correctional workers, and paramedics) are regularly exposed to potentially psychologically traumatic events that leave them vulnerable to occupational and posttraumatic stress injuries. In response, PSP organizations have developed and implemented peer support programs, as well as peer support apps, to promote health awareness among workers. This study was designed to evaluate the usability, usefulness, efficacy (i.e. self-reported mental health), and thoughts moving forward on whether to continue providing the PeerConnect app. A sample of PSPs (*N* = 455) from across 28 PSP organizations in Ontario, Canada, specifically 14 police services, 13 emergency services, and the Ontario provincial correctional service, participated in an online survey intended to explore PSPs’ views and experiences with the app. Of the 455 PSPs surveyed, 226 were PeerConnect users, and 229 were non-users. A series of *t*-tests and chi-square tests were conducted to compare users and non-users. Overall, the Connect feature was the most used by the PSPs. Among Connect users, 74.8% used the feature primarily to provide peer support, while 21.8% used the feature primarily to receive peer support. When assessing the usefulness of the app, 70.8% of app users were satisfied with the app, although only 40.3% of users found the app “extremely” or “very” useful in serving their well-being. When asked whether their organization should continue providing access to PeerConnect, 88.9% of users believed so. Findings demonstrate that PSPs who used PeerConnect were generally satisfied with the app and its features; however, there were barriers, such as a lack of internet access at work, that may have prevented non-users from using the app and highlighted the need for refinements in design, implementation, and integration into organizational culture. In response, we suggest public safety organizations consider finding innovative ways to continue to offer peer support opportunities for PSPs.

Police officers, correctional workers, firefighters, paramedics, and other public safety professionals (PSPs) witness a series of events that make them vulnerable to occupational and posttraumatic stress injuries.^1^ Due in part to organizational (e.g. staff shortages) and operational stressors (e.g. shift work), the prevalence of mental health disorders among individuals working in the public safety sector is remarkably higher than in the general Canadian population.^2,3^ Such impacts affect not only PSPs themselves, but also their families, putting strain on their marriages and relationships with their children,^4^ as well as their work performance and the communities they serve. Given the imperative need to support the mental health of PSPs, peer support programs have gained traction as a non-clinical healthcare intervention tool for employees within the public safety sector. The increase in peer support programs has been accompanied by the emergence and growth of “peer support” apps. Such mobile health apps are designed to facilitate peer support programs and promote health awareness. In other words, they are meant to connect peers with peer supporters and encourage connection and follow-up for those in need. Some of those apps are designed by a private initiative, while others, like PeerOnCall, an app developed by a team of researchers from Canada^5^—are financed by the government.

App developers, private or public, market health apps as occupationally targeted, cost-effective, and accessible intervention platforms. More practically, these apps are developed and implemented to respond to the limited availability of in-person treatment.^6^ Thus, governments and public safety organizations in Canada and elsewhere have invested in mobile health technology as a driver of peer support uptake among employees. For example, in 2021, the Ministry of the Solicitor General in Ontario, Canada, piloted PeerConnect, an app developed by First Response Mental Health, a Canadian-based technology firm, specifically for PSPs. The app was piloted by police, correctional, and emergency (i.e. paramedics) services, offering public safety organizations in Ontario the opportunity to test-drive the app as a potential tool to advance peer support programs and promote employee well-being. However, despite the growing popularity of peer support apps, there is limited evidence of their effectiveness in facilitating peer support use and raising health awareness. The benefits and limitations of mobile health apps to support employee well-being in the public safety sector have been overlooked from an empirical perspective. The present study fills a gap in health apps by analyzing the views and experiences of Ontario PSPs with PeerConnect. More specifically, the current study aimed to determine whether PeerConnect achieved the intended goal of facilitating peer support services and promoting well-being among PSPs.

## Health apps

Health apps have become a popular tool for tracking one's health and wellness. Smartphones often come with preinstalled health apps, encouraging users to track their health information, such as sleep patterns, physical activities, and the intake of nutritious food. With the increased focus on mental health, health app designers broadened their understanding of health and started developing offers that promoted mental health practices and resources.^6^ Mental health apps have become a source of mental health care globally as they are cost-effective and offer some alternative relief when one cannot access psychotherapy or counselling.^7^ They offer a variety of features such as mood tracking, therapeutic exercises, safety plan creation, access to resources, and mental health games.^8^ Many apps have also been designed for specific mental health concerns, including depression,^9–12^ anxiety,^9,13,14^ schizophrenia,^15–17^ bipolar disorder,^18–20^ eating disorders,^21,22^ and posttraumatic stress disorder (PTSD).^23–25^ Despite the fast-growing use of mental health apps, concerns remain.

There are currently no standards for mental health app quality control,^26,27^ and many mental health apps are unregulated, which is potentially unsafe, as they may compromise user privacy and provide users with inaccurate information, which may lead to harmful effects.^8,28–30^ For instance, a 2021 study by Parrish and colleagues has demonstrated that apps meant to support individuals dealing with suicidal ideation presented inconsistencies in the language used to communicate policies and resources related to suicide crises.^31^ Such inconsistencies can affect and compromise one's ability to obtain help, especially in a crisis. The effectiveness of mental health apps has also been a site of continued controversy. Although many apps claim effectiveness, such claims are yet to be substantiated by scientific clinical data.^32–35^ Scholars conducting systematic reviews and meta-analyses of the health app literature have demonstrated that only 10%–14% of the apps have clinically validated evidence (i.e. research done with a control group) of their effectiveness.^35–37^ In other words, most health apps are not reliable as a sole source of mental health treatment; however, they are often used as such. However, apps developed by government agencies designed to support the resiliency of military personnel are more likely to be clinically evaluated and produce positive outcomes.^38,39^

Despite issues involving health apps’ regulation, effectiveness, and responsibility over well-being, the app market has become specialized, catering to specific population groups, like youth^40–42^ and their parents,^12,43^ Indigenous people,^44–46^ and cancer patients,^47–49^ to name a few. App designers also target specific occupational categories such as medical doctors,^50,51^ apprentice workers,^52^ nonmedical essential workers (e.g. workers in food, transportation, and other crucial industries),^53^ military personnel and veterans,^38,39,54–59^ and PSPs.^60^ In fact, government investment in potential solutions to support PSPs’ mental health during the pandemic has led to a surge in apps designed for PSPs,^53^ despite uncertainty over these apps’ efficacy.^61^ Recently, health apps designed for PSPs have started to include a feature to support and facilitate peer support programs.

## Peer support programs

In general, the research on the effectiveness of peer support programs is conflicting. On the one hand, the literature says peer support programs are not effective in treating trauma and psychological issues. On the other hand, the literature says peer support programs help combat stigma. For instance, a study examining a Critical Incident Stress Management (CISM) peer support program found that when adhering to the proper guidelines, peer support programs are seen as helpful for providing tools and coping strategies for PSPs.^62^ Although PSPs in a peer support program that highly adhered to the model guidelines were less likely to screen positive for alcohol use disorder and generalized anxiety disorder compared to PSPs who did not participate in CISM, this was not the case for the other mental health disorders analyzed (i.e. major depressive disorder, panic disorder, PTSD, social anxiety disorder). Additionally, an evaluation of Before Operational Stress, a yearlong resiliency program with integrated peer support aspects, revealed improvements in relationships with family and coworkers.^63^ Although there were trends of positive impacts on PSPs’ mental health disorder symptoms, there were only statistically significant improvements in PTSD symptoms. Despite the lack of evidence supporting mental health disorder improvement, the importance of reducing stigma and additional benefits of peer support programs, such as mental health literacy and self-efficacy,^64–66^ should not be disregarded.

A potential reason for the lack of evidence of psychological improvements among those enrolled in peer support programs is the inconsistency in methodologies and implementation.^67^ Currently, there are no national standards or guidelines for practicing peer support.^68^ Further, the definitions conceptualizing “peer” and “peer support” differ between organizations. To illustrate, Price et al.,^68^ in their study using a document analysis, found that there were three models for conceptualizing peer support: peer-enabled, peer-led, and peer-partnership. Moreover, peer support was implemented through two delivery methods: programs and services. Their research exemplifies the need for a typology of peer support to inform future research.

## Peer support apps

Peer support apps have been created for people living with cancer^69^; specific mental health concerns such as major depressive disorder, bipolar disorder, and schizophrenia^70–74^; and people who self-harm.^75,76^ However, to our knowledge, there is limited literature available on peer support apps specifically for PSPs. These studies include the qualitative follow-up study to the current study, which found that PSPs used the peer support feature of the app the most out of all features, and they appreciated the convenience of the PeerConnect app and how it helped reduce stigma surrounding peer support,^60^ and a study exploring how organizational culture may impact implementation of PeerOnCall and PeerOnCall Support.^77^ Beyond this, research is available on how health apps have been used to enhance peer support programs. For instance, CrewCare, a mental health app for first responders to help them cope with traumatic events, includes self-assessment and resource sections.^78^ The resources include a list of peer support team members that the first responders can contact. One participant in this study indicated that they would like peer support to be included in the app, as it would allow for an anonymous way of reaching out for help. Another study examined a physical activity intervention for first responders and their partners using a Fitbit tracking device and app to track their health, and a Facebook group to deliver the 10-week program, including sharing pictures and posts.^79^ Program participants indicated that they highly valued the peer support aspect of the program, even forming friendships outside the program. Additionally, a scoping literature review of health apps for PSPs and military personnel found that mental health apps that included gaming and social aspects resulted in more positive outcomes and an increase in perceived peer support.^39^ Such findings show promise for peer support apps for PSPs.

### PeerConnect

PeerConnect, the app under consideration in our present study, includes five features: four custom features created by the app developer and one feature developed by the Canadian Institute for Public Safety Research and Treatment (CIPSRT). The custom features are Connect, Newsfeed, Resources, and Events. Connect is the app's main feature, allowing PSPs to access peer support by “pushing a button.” Newsfeed and Resources offer employers (i.e. the services) the opportunity to distribute relevant news and information to app users. The Events feature allows employers to advertise relevant events to app users. Finally, the Self-Assessment feature, developed by CIPSRT, links app users to publicly available online screening tools that help identify mood disorders, anxiety disorders, or PTSD symptoms.

## Present study

This study was commissioned by the Ministry of the Solicitor General of Ontario's Mental Health Secretariat to determine whether PeerConnect was effective and warranted continued budget allocation. Thus, we designed a mixed-method research project to survey (*N* = 455) and interview (*N* = 23) PSPs working for the agencies piloting PeerConnect. While the survey enabled us to gather broad, quantifiable, and generalizable data from participating services, including those in remote areas of the province, the interviews provided context and nuance to supplement the survey findings. However, both the survey and the interviews were intended to explore the app's “usability” and “usefulness.” Usability refers to the degree to which the app is fit to be used. Usefulness refers to whether PSPs, particularly police, correctional, and emergency services employees, believed the app was relevant to them. Thus, we explored factors that may encourage and discourage the use of health apps, such as lack of time, internet fatigue, privacy concerns, and disbelief in employers and their initiatives regarding employee well-being. We also inquired about participants’ opinions on whether the services should continue to provide PeerConnect. Finally, we characterized app users and non-users in terms of occupation and demographics and analyzed their views of the app. The article provides insights into the usability and use of health apps designed to facilitate peer support for PSPs. Given the broad scope of the project, the present article focuses exclusively on the survey findings.

## Methods

### Data collection

The survey for the present study targeted PSPs within services that were piloting PeerConnect. A total of 18,800 app licenses or potential app users came from 44 services, including 23 police services, 20 emergency services, and the provincial correctional service in Ontario, Canada. The research team contacted all organizations, inviting them to participate in the app evaluation, and 28 organizations (14 police services, 13 emergency services, and the provincial correctional service) participated. These organizations held 16,300 app licenses, accounting for about 87% of the population eligible to use the app. The survey instrument comprised 56 questions organized into 13 thematic blocks. These blocks addressed: participation consent; employer information; general questions about PeerConnect; individual sections on each app feature (Newsfeed, Resources, Events, Self-Assessment, and Connect); participants’ overall evaluation of the app; questions for those identified as non-users; demographic information; and follow-up interview participation. The research team developed the survey questions in partnership with the Ministry of the Solicitor General, which was designed with the intent of capturing user perspectives on the app and focuses on gathering practical insights regarding usability and utility of PeerConnect. Thus, we did not have the option of using validated scales because none such exist on such a nuanced and specific application.

To ensure that survey participants had the time to try the app and to increase the quality of research data, the services had to have used the app for at least three months at the beginning of data collection. The survey was delivered using the online platform, Qualtrics. The participating services assisted with survey distribution by internally emailing their service employees with the survey link, as well as sending out reminder emails. The survey link was active between 14 February and 18 April 2022, and took participants ∼8 minutes on average to complete the survey. The research's ethics protocols received approval from the Memorial University of Newfoundland's research ethics board (no. 20220020).

### Participants

A total of 485 individuals participated in the survey. However, after cleaning the data to remove participants who declined to participate after reading the consent form and eliminating participants with incomplete surveys (i.e. those who stopped partway or submitted the survey with unanswered questions), the final sample included 455 participants.

Of the 455 participants, almost half (49.5%) worked in emergency services, 27.3% worked in correctional services, and 21.8% worked in police services. Seven participants (1.5%) did not disclose their service type. Of the 455 participants, 226 (49.7%) answered “yes” to the question “Do you use the app PeerConnect?” and were thus categorized as “app users,” while 229 (50.3%) answered “no” and were categorized as “non-users.” In terms of gender, 233 (51.2%) self-identified as women, 208 (45.7%) self-identified as men, 1 (0.2%) self-identified as other, and 13 (2.9%) did not disclose their gender. On average, participants were 41.8 years of age (*SD* = 9.8), ranging from 16 to 67 years. Nearly three-quarters (72.7%) of participants were married or in a common-law relationship; 11.4% had never married; 10.8% were separated or divorced; 0.2% were widowed; and 4.8% did not disclose their marital status. Over half of the participants (53.8%) had a college or technical diploma, while about 36.3% had a university certificate or degree at a bachelor's level or above. A small minority had either a university certificate or diploma below the bachelor's level (4.4%) or a high-school diploma (3.7%), and 1.8% did not report their highest degree of education. On average, participants worked ∼42.4 hours per week (*SD* = 8.8), ranging from 4 to 84 hours per week.

### Survey

The survey instrument consisted of 56 multiple-choice questions. We examined the demographic information to compare app users versus non-users. For the purposes of the present study, we included data related to app usability, usefulness, efficacy (i.e. self-reported health), and thoughts moving forward on whether to continue providing the app.

To assess the app's usability for app users, the survey inquired into the following topics: (1) frequency users engaged with the app and its features; (2) users’ role when using the Connect feature (i.e. peer support provider or receiver); and (3) obstacles when using the app. Both users and non-users were asked about the obstacles hindering app usability, such as knowledge of online technology and internet access and availability of technology. Non-users were also asked whether they had been invited to use the app to enable an examination of the association between non-use and the app's promotional activities. Notably, the Ministry that commissioned the project expressed concerns about whether all employees in the agencies where the app was piloted had, in fact, received an invitation from their employers to try it.

To assess the app's usefulness for app users, the survey inquired into the following topics: (1) whether app users were satisfied with the app and its features; and (2) whether app users found the app useful in serving their well-being. To provide insights into the reasons underpinning non-users’ choice for not using PeerConnect, the survey inquired into the following topics: (1) if non-users had used the app before and if they would use it again; and (2) if non-users used any health app beyond PeerConnect.

To assess whether using the app impacts mental health, the survey included a question examining participants’ self-reported mental health status in the last three months. Responses included “excellent,” “good,” “average,” “poor,” and “very poor.” If participants were unsure of their mental health status, they were given the option to select “I don’t know.” Additionally, participants were given the option to select “prefer not to say.”

To support policymaking involving health apps, the survey queried participants about their opinion on the following topics: (1) health apps in general (i.e. if apps can facilitate well-being and contribute to health awareness); (2) peer support with and without an app; (3) peer support outcomes (i.e. feelings associated with peer support); (4) feelings towards employers’ wellness initiatives; (5) ability to engage in self-care at work; and (6) factors that can encourage and discourage the use of health apps (i.e. lack of time, internet fatigue, and privacy concerns). All participants were asked whether they believed an app could support their well-being. Finally, PeerConnect users were asked whether their organization should continue providing access to the app.

### Statistical analysis

We analyzed data using IBM SPSS Statistics (Version 29.0.1.1) and Jamovi (Version 2.6.26). Descriptive statistics (frequencies, percentages, means, and standard deviations) were computed to summarize demographic variables and survey responses. To compare app users and non-users on continuous variables (e.g. age and self-reported mental health scores), we used Welch's *t*-tests, which adjust for unequal variances. For categorical variables (e.g. service type, internet access, and perceptions of health apps), we conducted Pearson chi-square (χ²) tests of independence. Where significant χ² results were observed, Cramer's *V* was reported as a measure of effect size. For *t*-tests, Cohen's *d* was calculated to interpret the magnitude of differences. All statistical tests were two-tailed, and a *p*-value < .05 was considered statistically significant.

## Results

### App users versus non-users

Users and non-users had similar demographic profiles; however, there were statistically significant differences. A Welch's *t*-test was used to determine whether there was a difference in the age of users and non-users. App users (*M* = 40.8, *SD* = 8.8) were significantly younger in age than non-users (*M* = 42.7, *SD* = 10.6), *t*(418.779) = −1.981, *p* = .048, Cohen's *d* = −0.190. A chi-square test was used to determine whether there was a difference in the service type of users and non-users, χ^2^(2, *N* = 455) = 49.878, *p* < .001, Cramer's *V* = 0.334. When examining service type, most app users (61.5%) worked at emergency services, followed by police services (25.2%) and correctional services (12.8%). Meanwhile, most non-users worked in correctional services (41.5%), followed by emergency services (37.6%) and police services (18.3%). There were no other significant group differences in terms of demographics (*p* > .05).

### Usability

To assess the app's usability, we first examined the frequency users engaged with it and its features. When assessing the frequency of overall app usage, 2.7% of participants reported using the app daily, 15.9% two to four times a week, 15.9% once a week, 9.7% once every 2 weeks, and 16.8% once a month. However, almost a third (32.7%) of participants rarely use the app, an additional 2.7% did not know how often they use the app, and 3.5% preferred not to say how often they use the app.

Connect, the app's most important feature, was the most used feature (47.3%), followed by Newsfeed (20.8%), Resources (15.0%), Self-Assessment (3.1%), and Events (0.4%). Based on information from health liaisons at the organizations, the Events feature was not “much” promoted due to the restrictions imposed by the COVID-19 pandemic on in-person gatherings. Thus, the pandemic may have affected Events’ usability. Among app users, 52.7% used Connect, the app's primary feature. Nevertheless, a large group (45.6%) had never used it. Four app users (1.8%) preferred not to say if they used the Connect feature. For a more detailed breakdown of the frequency of individual feature usage for Newsfeed, Resources, and Events, see Table 1.

**Table 1.** Frequency of usage for Newsfeed, Resources, and Events among PeerConnect users.

	Newsfeed (%)	Resources (%)	Events (%)
At least once a week	22.1	10.2	6.7
Once every 2 weeks	11.5	7.1	4.9
Once a month	18.1	17.7	7.5
Rarely	28.8	35.4	30.1
Never	17.7	24.3	47.3
This feature is not available on my app	0.0	0.9	1.8
Don’t know	0.9	2.7	0.9
Prefer not to say	0.9	1.8	0.9

*N* = 226.

Next, we examined users’ roles when using the Connect feature (i.e. peer support provider or receiver), as well as how often they sought peer support through the Connect feature. Most Connect users (74.8%) used the feature primarily to provide peer support, while 21.8% used the feature primarily to receive peer support. Four Connect users (3.4%) preferred not to say their primary role. Approximately half of Connect users (48.7%) sought peer support just once, 10.9% sought peer support twice, 0.8% sought peer support three times, 5.0% sought peer support four times, 3.4% sought peer support five times, 0.8% sought peer support eight times, 0.8% sought peer support 10 times, and 10.1% sought peer support more than 10 times. About 9.2% did not know how many times they sought peer support, while 10.1% preferred not to say how many times they sought peer support.

Participants were asked a variety of questions about potential obstacles to app usage. Some of the obstacles explored were external to the app, including knowledge of online technologies, internet access, and internet access when working in remote areas, while others were related to the app, such as technical difficulties (i.e. “bugs”) to download, use the app, and privacy concerns. When looking at participants’ knowledge of online technology, there were no differences found between users and non-users in terms of knowledge of online technology, χ^2^(4, *N* = 453) = 7.075, *p* = .132, Cramer's *V* = 0.125. Most participants reported having excellent knowledge (18.1% of users and 19.2% of non-users), good knowledge (39.4% of users and 37.6% of non-users), or average knowledge (39.8% of users and 36.7% of non-users). Few participants rated their knowledge of online technologies as poor (1.8% of users and 5.7% of non-users) or very poor (0% of users and 0.9% of non-users). Few users (0.9%) preferred not to say their knowledge of online technology. We tested whether there were differences between users and non-users in how often they have access to the internet while at work. The results, shown in Table 2, show that the differences involving users and non-users and internet access at work were statistically significant, χ^2^(4, *N* = 452) = 15.119, *p* = .004, Cramer's *V* = 0.183. We also tested whether there were differences between users and non-users in how often they work in remote areas with limited internet access. The results, shown in Table 2, show that the differences involving users and non-users and how often they work in remote areas were statistically significant, χ^2^(4, *N* = 452) = 10.939, *p* = .027, Cramer's *V* = 0.156.

**Table 2.** Obstacles external to the app experienced by PeerConnect users and non-users.

	Internet access at work (%)		Working in remote areas (%)	
	Users	Non-users	Users	Non-users
Always	48.2	35.4	2.7	1.3
Frequently	33.6	33.6	9.7	7.4
Occasionally	13.7	20.5	22.6	21.0
Rarely	2.7	8.7	39.8	32.3
Never	0.9	1.3	23.9	38.0
Prefer not to say	0.9	0.4	1.3	0.0

*N*_users_ = 226 and *N*_non-users_ = 229.

App users were asked if they found PeerConnect easy to download. The majority (90.3%) of participants found the app easy to download, 7.5% found the app somewhat easy to download, 1.8% did not find the app easy to download, and 0.4% preferred not to say. App users were also asked whether they found PeerConnect easy to use. Nearly two-thirds (61.5%) of app users found the app easy to use, 31.9% found the app somewhat easy to use, 4.4% did not find the app easy to use, 1.3% did not know if they found the app easy to use, and 0.9% preferred not to say. App users were asked whether they had any privacy concerns when using PeerConnect. Most participants (85.8%) had no privacy concerns, 8.0% had privacy concerns sometimes, 3.1% had privacy concerns, 2.2% were unsure if they had privacy concerns, and 0.9% preferred not to say. Non-users were asked if they were invited to use the app. Based on the survey results, only 21.4% of non-users were not invited to use the app, suggesting they may not have opted out of using it voluntarily. The remaining non-users were either asked (59.7%) or could not remember if they were asked (17.0%), and 1.9% preferred not to say if they were invited to use the app.

### Usefulness

To assess the app's usefulness for app users, we first examined whether app users were satisfied with the app and its features. The results, shown in Table 3, show that many participants (70.8%) were satisfied with the app. App users were the most satisfied (either “extremely satisfied” or “somewhat satisfied”) with the features Connect (87.8%), Self-Assessment (73.2%), Resources (59.8%), Newsfeed (57.5%), and Events (27.8%), in this order (see Table 3).

**Table 3.** Satisfaction with the app and its features among PeerConnect users.

	App (%)	Connect (%)	Newsfeed (%)	Resources (%)	Self-Assessment (%)	Events (%)
Extremely satisfied	26.1	55.3	14.0	14.8	22.5	6.1
Somewhat satisfied	44.7	32.5	43.5	45.0	50.7	21.7
Neither satisfied nor dissatisfied	20.4	5.7	26.3	21.9	9.9	38.3
Somewhat dissatisfied	3.5	2.4	1.6	1.8	1.4	0.0
Extremely dissatisfied	0.4	0.0	1.1	0.0	0.0	0.9
Don’t know	4.4	0.8	13.0	15.4	12.7	27.8
Prefer not to say	0.4	3.3	1.0	1.2	2.8	5.2
*N*	226	123	186	169	71	115

Next, we examined whether app users found the app and its features useful in serving their well-being. The results, shown in Table 4, show that only 40.3% of users found the app “extremely” or “very” useful in serving their well-being. Most users found Connect (69.1%) and Self-Assessment (47.9%) particularly useful (i.e., “extremely useful” or “very useful”) for their well-being. Meanwhile, Resources (29.6%), Newsfeed (24.2%), and Events (10.4%) were considered the least conducive to promoting user well-being (see Table 4).

**Table 4.** Usefulness of the app in serving PeerConnect users’ well-being.

	App (%)	Connect (%)	Newsfeed (%)	Resources (%)	Self-Assessment (%)	Events (%)
Extremely useful	12.4	38.2	4.3	7.1	16.9	3.5
Very useful	27.9	30.9	19.9	22.5	31.0	7.0
Moderately useful	28.8	12.2	36.6	29.0	32.4	20.0
Slightly useful	17.7	8.9	19.4	14.8	4.2	20.9
Not at all useful	3.5	1.6	4.3	1.2	0.0	8.7
Don’t know	9.3	4.1	14.0	23.7	12.7	37.4
Prefer not to say	0.4	4.1	1.6	1.8	2.8	2.6
*N*	226	123	186	169	71	115

To gain insights into any potential rejection of PeerConnect, we examined whether non-users had used the app before and if they would use it again. Most non-users (88.2%) had not used the app, while 10.0% had used the app, and 1.7% preferred not to say. A further breakdown of the 10% of those who had used the app, 1.7% would use it again, 3.9% would maybe use it again, 3.5% would not use it again, and 0.9% did not know if they would use it again. Additionally, non-users were asked whether they have ever used other health apps to help their mental health. Most non-users (82.5%) did not use any health app, while only 16.6% used a health app but not PeerConnect. Two participants (0.9%) preferred not to say whether they used any health apps.

Both users and non-users were asked whether they believed an app could support their well-being. Nearly half of all participants (44.8%; 63.3% of users and 26.6% of non-users) said they believed an app could support their well-being, 35.6% (28.3% of users and 42.8% of non-users) believed that an app may be able to support their well-being, 14.5% (6.2% of users and 22.7% of non-users) did not believe that an app could support their well-being, 4.8% (1.8% of users and 7.9% of non-users) were unsure if an app could support their well-being, and one user (0.4%) preferred not to say. A chi-square test revealed significant differences between users and non-users, χ^2^(2, *N* = 432) = 61.777, *p* < .001, Cramer's *V* = 0.378.

### Efficacy

To examine whether there is a relationship between PeerConnect use and mental health, we compared app users and non-users on their self-reported mental health status in the last 3 months. The average mental health score for users (*M* = 3.12, *SD* = 0.951, *SEM* = .064) was higher than for non-users (*M* = 2.88, *SD* = 1.067, *SEM* = .071). A Welch's *t*-test analysis revealed that the average self-reported mental health score of users and non-users was significantly different, *t*(441.121) = 2.431, *p* = .015, Cohen's *d* = 0.230.

### General views on health apps and peer support

All survey participants, including PeerConnect users and non-users, were invited to give their opinions on eight statements that could speak to the overall usefulness of health apps in the public safety sector (see Table 5). Such statements included: “I prefer seeking peer support from a trusted co-worker without using an app” (statement 1); “Health apps remind me to care for my well-being” (statement 2); “I feel supported when someone at work shows they care about my well-being” (statement 3); “I have time to practice self-care during my shift” (statement 4); “I don’t have time to access the internet during my shift” (statement 5); “I avoid using health apps because I have internet fatigue” (statement 6); “I believe employers use apps like the PeerConnect app to spy on employees” (statement 7); and “I believe my organization genuinely cares about my mental health” (statement 8). When comparing users and non-users on their opinions on health apps, there were significant differences. Users reported being in more agreeance with statements 2–4 and 8 (χ^2^(4, *N* = 434) = 49.609, *p* < .001, Cramer's *V* = 0.338; χ^2^(4, *N* = 451) = 50.648, *p* < .001, Cramer's *V* = 0.335; χ^2^(4, *N* = 450) = 24.693, *p* < .001, Cramer's *V* = 0.234; χ^2^(4, *N* = 450) = 49.512, *p* < .001, Cramer's *V* = 0.332; respectively). Non-users reported being in more agreeance with statements 1 and 5–7 (χ^2^(4, *N* = 437) = 28.176, *p* < .001, Cramer's *V* = 0.254; χ^2^(4, *N* = 450) = 68.074, *p* < .001, Cramer's *V* = 0.389; χ^2^(4, *N* = 440) = 36.443, *p* < .001, Cramer's *V* = 0.288; χ^2^(4, *N* = 426) = 44.478, *p* < .001, Cramer's *V* = 0.323; respectively).

**Table 5.** Overall usefulness of health apps among PeerConnect users and non-users.

	Strongly agree (%)	Agree (%)	Neutral (%)	Disagree (%)	Strongly disagree (%)	Don’t know (%)	Prefer not to say (%)
Statement 1	18.5	27.0	34.3	10.8	5.5	2.2	1.8
Statement 2	5.9	35.6	30.1	18.5	5.3	4.4	0.2
Statement 3	30.8	53.6	8.4	3.1	3.3	0.7	0.2
Statement 4	2.9	18.0	21.3	29.7	27.0	0.9	0.2
Statement 5	11.2	14.7	18.7	32.1	22.2	0.4	0.7
Statement 6	4.0	15.2	25.7	39.8	12.1	2.9	0.4
Statement 7	3.1	5.1	8.8	37.1	39.6	5.7	0.7
Statement 8	8.1	25.9	22.6	18.0	24.2	0.9	0.2

*N* = 455.

Finally, PeerConnect users were asked whether their organization should continue providing access to the app. A large majority (88.9%) thought their organizations should continue providing access to PeerConnect, while only 2.7% did not think that their organizations should continue providing the app. An additional 7.5% were unsure, and 0.9% preferred not to say.

## Discussion

### Principal findings

We surveyed 455 PSPs, 226 of whom were users and 229 of whom were non-users of the PeerConnect app. Overall, app users were satisfied with the app and its features, finding them useful in serving their well-being, and therefore believing that their organizations should continue providing access to the app. Conversely, non-users do not believe that PeerConnect or any health app can help support their well-being, indicating their disinterest in any health app.

Connect was the most used feature offered by PeerConnect, with 47.3% of app users reporting using the app the most of the five features, and 52.7% of app users reporting having ever used the app. The next most popular feature was Newsfeed, followed by Resources. Such findings align with our follow-up qualitative interview.^60^ Among Connect users, 74.8% used the feature primarily to provide peer support; however, that does not mean that they did not additionally seek peer support, but rather that the app is useful in assisting with peer support programs. Surprisingly, 45.6% of app users had never used the Connect feature despite it being the main feature of PeerConnect. Perhaps, like app non-user reports from the qualitative study, app users who did not use the Connect feature may not believe that peers are adequately trained to provide peer support,^60^ which, given the lack of guidelines and standards surrounding practicing peer support, is understandable.^68^ Nevertheless, why app users never used the Connect feature requires additional exploration to determine if they failed to do so due to a lack of need, a lack of interest, or if they simply wanted the feature for others to use rather than themselves. This may be connected to how 12.4% of the sample overall found the app to be extremely helpful, suggesting that more research into what would be helpful for peer support is warranted.

In terms of usefulness, most users were satisfied with the app (70.8%), although only 40.3% found it “extremely” or “very” useful in serving their well-being. When looking at user satisfaction with and usefulness in supporting well-being in terms of the app's features, app users were most satisfied with Connect, followed by Self-Assessment, Resources, Newsfeed, and Events for both. As the study occurred during the COVID-19 pandemic, it is unsurprising that the Events feature was the feature users were the least satisfied with, as was found in Foley and Ricciardelli.^60^ Both users and non-users were asked various questions about potential obstacles to using the app. Most PeerConnect users found the app easy or somewhat easy to download (90.3%), and most (85.8%) had no privacy concerns when using the app. When looking at users’ and non-users’ obstacles outside of the app that may hinder PeerConnect usage, users reported having more access to the internet at work compared to non-users. However, there were no differences in terms of knowledge of online technologies, and non-users reported working in remote areas with limited internet access *less* than app users. Looking closer at non-users, although 21.4% reported not having been invited to use the app, suggesting that they involuntarily opted out of using it, many (59.7%) were invited. When asked if non-users had ever used the app, only 10.0% reported having ever used it. Likewise, only 16.6% reported ever having used any health app besides PeerConnect. To further support this sentiment, non-users were much less likely to believe that an app could support their well-being. Non-users also reported less agreeance with statements supporting the use of health apps in general, such as preferring to seek peer support without the use of an app and believing that health apps provided by employers are used to spy on employees. As such, perhaps non-users do not necessarily dislike or are disinterested in PeerConnect itself, but rather health apps in general and would not use PeerConnect whether given the opportunity or not.

One of the main goals of PeerConnect is to support PSPs’ mental health and well-being by reducing barriers to support seeking. Therefore, we anticipated and found users self-reported a higher average mental health score over the last three months compared to non-users. Although we cannot say that PeerConnect itself is the cause of any improvement in mental health (i.e. the app is a vehicle for support, not an intervention or treatment), we do consider likely that those using PeerConnect are more aware of their mental health and have sought additional means of supporting their mental health. Another potential explanation is that users feel more supported by their employers. For instance, when asked about general views on health apps and peer support, app users reported agreeing more with the statements “I feel supported when someone at work shows they care about my well-being” and “I believe my organization genuinely cares about my mental health.” When examining demographic differences between users and non-users, most non-users were older and worked in correctional services, while users were younger, with most working in emergency services. In terms of age, younger PSPs may be more willing to use technology such as health apps to support their mental health compared to older PSPs, like those in the general population.^80^ Previous research on PSPs health knowledge, stigma, and service use has found that correctional workers reported the most mental health knowledge, the least amount of mental health stigma, and the highest intentions of using mental health services.^81,82^ Possibly, rather than the occupation, the specific organizations for which non-users work affect their use of PeerConnect. The app was promoted by the organizations and management, and correctional workers have reported concerns over organizational stressors, such as relationships, which can be strained, with management, and different levels of mental health support within their organizations.^83^ No matter the service type, PSP organizations, in addition to continuing to provide access to health apps, must continue to work on promoting a more supportive and caring work environment.

The findings of our study highlight the advantages and drawbacks of PeerConnect. The findings complement those of Foley and Ricciardelli,^60^ which found both users and non-users to believe that PeerConnect helps raise awareness of wellness and potentially reduce stigma, data which we were not able to capture in the quantitative survey. Like app users in the current study who found the app useful, users in the qualitative study appreciated how convenient the app and the app's features were, especially peer support providers who found Connect particularly useful in facilitating peer support services, potentially explaining why so many PeerConnect users in both studies were peer support providers. Together, both studies demonstrate the potential benefits and limitations of PeerConnect, providing the Ministry of the Solicitor General with information to make an informed decision regarding adoption and development.

### Limitations

There are limitations to the current study. First, this research offers a somewhat positive view of PeerConnect due to participant bias. About 75% of Connect users used the feature as peer support providers, meaning they were members of peer support programs at their services. Thus, the users’ views captured in the survey represent primarily the view of peer supporters who tend to be favorable to initiatives that promote mental health and wellness, rather than the opinions of employees using the app to access peer support services, the population for which the app was created and adopted. Second, several factors limited research participation and sample size. In particular, the lack of support for the app at the service level, uncertainty about funding to support the app in the future, workload (i.e., data collection overlapped to some extent with social movements in Ontario), and delays in the app deployment prevented all services piloting the app from participating in the survey. Although the sample was smaller than intended, the sample was large enough to yield statistical significance. Additionally, there was limited information provided on specific features. For example, the use of the Events feature was compromised by the COVID-19 pandemic, which limited public gatherings and thus the use of Events. Finally, our study did not use a validated measure of mental health to capture participants’ well-being, as the goal of our study was not to measure mental health; instead, we were measuring how well the PeerConnect app worked as a vehicle to connect people to others for support. However, future research should focus on a study of peer support as a mental health intervention that uses validated mental health measures.

## Conclusion

Our study explored PeerConnect through a quantitative survey. Overall, app users found PeerConnect to be useful in supporting their mental health and well-being, finding the Connect feature especially useful. However, many participants did not use the app for various reasons, including not believing that health apps can support well-being and preferring to seek peer support without the help of an app. PSP organizations must continue to make efforts in supporting employee well-being, including continuing to develop and offer peer support apps. Future research must continue to evaluate mental health and peer support apps for both PSPs, especially with a focus on occupational subgroups and gender, as well as the general population. By providing one of the first cross-sector evaluations of a peer support app for PSPs, our study offers critical insights that can guide evidence-informed program development, inform policy decisions, and ultimately enhance the mental health and resilience of those who serve our communities.

## Supplemental Material

sj-docx-1-dhj-10.1177_20552076251384142 - Supplemental material for Views on the usability and usefulness of the PeerConnect app among Ontario public safety professionals Supplemental material, sj-docx-1-dhj-10.1177_20552076251384142 for Views on the usability and usefulness of the PeerConnect app among Ontario public safety professionals by Gillian Foley, Marcella Cassiano and Rosemary Ricciardelli in DIGITAL HEALTH

## References

[1] Oliphant RC . Healthy minds, safe communities: supporting our public safety officers through a national strategy for operational stress injuries. Standing Committee on Public Safety and National Security October 2016: 1–39. https://www.ourcommons.ca/Content/Committee/421/SECU/Reports/RP8457704/securp05/securp05-e.pdf.

[2] Carleton RN Afifi TO Turner S , et al. Mental disorder symptoms among public safety personnel in Canada. Can J Psychiatry 2018; 63: 54–64. 28845686 10.1177/0706743717723825 PMC5788123

[3] Carleton RN Afifi TO Taillieu T , et al. Assessing the relative impact of diverse stressors among public safety personnel. Int J Environ Res Public Health 2020; 17: 1234. 32075062 10.3390/ijerph17041234 PMC7068554

[4] Ricciardelli R Carleton RN Groll D , et al. Qualitatively unpacking Canadian public safety personnel experiences of trauma and their well-being. Canadian Journal of Criminology and Criminal Justice 2018; 60: 566–577.

[5] PeerOnCall . PeerOnCall App, https://www.oncallapp.ca/app/ (2025, accessed 14 April 2025).

[6] Chandrashekar P . Do mental health mobile apps work: evidence and recommendations for designing high-efficacy mental health mobile apps. mHealth 2018; 4: 6–6. 29682510 10.21037/mhealth.2018.03.02 PMC5897664

[7] Becker S Miron-Shatz T Schumacher N , et al. Mhealth 2.0: experiences, possibilities, and perspectives. JMIR mHealth uHealth 2014; 2: e24. 10.2196/mhealth.3328 PMC4114478 25099752

[8] Giota KG Kleftaras G . Mental health apps: innovations, risks and ethical considerations. ETSN 2014; 03: 19–23.

[9] Linardon J Messer M Goldberg SB , et al. The efficacy of mindfulness apps on symptoms of depression and anxiety: an updated meta-analysis of randomized controlled trials. Clin Psychol Rev 2024; 107: 102370. 38056219 10.1016/j.cpr.2023.102370 PMC10872959

[10] Luo Y Stice BL Lenz AS . Mental health apps for depression: a meta-analysis. J Counseling Develop 2025; 103: 25–38.

[11] Polhemus A Simblett S Dawe-Lane E , et al. Health tracking via mobile apps for depression self-management: qualitative content analysis of user reviews. JMIR Hum Factors 2022; 9: e40133. 10.2196/40133 PMC9730209 36416875

[12] Sprenger M Mettler T Osma J . Health professionals’ perspective on the promotion of e-mental health apps in the context of maternal depression. PLoS ONE 2017; 12: e0180867. 10.1371/journal.pone.0180867 PMC5507525 28704442

[13] Balaskas A Schueller SM Cox AL , et al. The functionality of mobile apps for anxiety: systematic search and analysis of engagement and tailoring features. JMIR mHealth uHealth 2021; 9: e26712. 10.2196/26712 PMC8529472 34612833

[14] Hammond TE Lampe L Campbell A , et al. Psychoeducational social anxiety mobile apps: systematic search in app stores, content analysis, and evaluation. JMIR mHealth uHealth 2021; 9: e26603. 10.2196/26603 PMC8493451 34546179

[15] Ben-Zeev D Brenner CJ Begale M , et al. Feasibility, acceptability, and preliminary efficacy of a smartphone intervention for schizophrenia. Schizophr Bull 2014; 40: 1244–1253. 24609454 10.1093/schbul/sbu033 PMC4193714

[16] Chen HH Hsu HT Lin PC , et al. Efficacy of a smartphone app in enhancing medication adherence and accuracy in individuals with schizophrenia during the COVID-19 pandemic: randomized controlled trial. JMIR Ment Health 2023; 10: e50806. 10.2196/50806 PMC10727482 38096017

[17] Firth J Torous J . Smartphone apps for schizophrenia: a systematic review. JMIR mHealth uHealth 2015; 3: e102. 10.2196/mhealth.4930 PMC4704940 26546039

[18] Beiwinkel T Kindermann S Maier A , et al. Using smartphones to monitor bipolar disorder symptoms: a pilot study. JMIR Ment Health 2016; 3: e2. 10.2196/mental.4560 PMC4720836 26740354

[19] Nicholas J Larsen ME Proudfoot J , et al. Mobile apps for bipolar disorder: a systematic review of features and content quality. J Med Internet Res 2015; 17: e198. 10.2196/jmir.4581 PMC4642376 26283290

[20] Pahwa M McElroy SL Priesmeyer R , et al. KIOS: a smartphone app for self-monitoring for patients with bipolar disorder. Bipolar Disord 2024; 26: 84–92. 37340215 10.1111/bdi.13362 PMC10730767

[21] Chiang CP Hayes D Panagiotopoulou E . Apps targeting anorexia nervosa in young people: a systematic review of active ingredients. Transl Behav Med 2023; 13: 406–417. 36753537 10.1093/tbm/ibad003 PMC10255767

[22] Lindgreen P Lomborg K Clausen L . Patient experiences using a self-monitoring app in eating disorder treatment: qualitative study. JMIR mHealth uHealth 2018; 6: e10253. 10.2196/10253 PMC6035344 29934285

[23] Davis CA Miller M McLean CP . The impact of user engagement with exposure components on posttraumatic stress symptoms in an mHealth mobile app: secondary analysis of a randomized controlled trial. JMIR mHealth uHealth 2024; 12: e49393–e49393. 10.2196/49393 PMC11269958 39036876

[24] Owen JE Jaworski BK Kuhn E , et al. mHealth in the wild: using novel data to examine the reach, use, and impact of PTSD coach. JMIR Ment Health 2015; 2: e7. 10.2196/mental.3935 PMC4607374 26543913

[25] Rodriguez-Paras C Tippey K Brown E , et al. Posttraumatic stress disorder and mobile health: app investigation and scoping literature review. JMIR mHealth uHealth 2017; 5: e156. 10.2196/mhealth.7318 PMC5680516 29074470

[26] Neary M Schueller SM . State of the field of mental health apps. Cogn Behav Pract 2018; 25: 531–537. 33100810 10.1016/j.cbpra.2018.01.002 PMC7584258

[27] Torous J Andersson G Bertagnoli A , et al. Towards a consensus around standards for smartphone apps and digital mental health. World Psychiatry 2019; 18: 97–98. 30600619 10.1002/wps.20592 PMC6313231

[28] Huckvale K Nicholas J Torous J , et al. Smartphone apps for the treatment of mental health conditions: status and considerations. Curr Opin Psychol 2020; 36: 65–70. 32553848 10.1016/j.copsyc.2020.04.008

[29] Koh J Tng GYQ Hartanto A . Potential and pitfalls of Mobile mental health apps in traditional treatment: an umbrella review. JPM 2022; 12: 1376. 36143161 10.3390/jpm12091376 PMC9505389

[30] Terry NP Gunter TD . Regulating mobile mental health apps. Behav Sci Law 2018; 36: 136–144. 29659069 10.1002/bsl.2339

[31] Parrish EM Filip TF Torous J , et al. Are mental health apps adequately equipped to handle users in crisis? Crisis 2022; 43: 289–298. 34042465 10.1027/0227-5910/a000785 PMC8641126

[32] Larsen ME Nicholas J Christensen H . Quantifying app store dynamics: longitudinal tracking of mental health apps. JMIR mHealth uHealth 2016; 4: e96. 10.2196/mhealth.6020 PMC4995352 27507641

[33] Larsen ME Huckvale K Nicholas J , et al. Using science to sell apps: evaluation of mental health app store quality claims. npj Digit Med 2019; 2: 18. 31304366 10.1038/s41746-019-0093-1 PMC6550255

[34] van der Meer CAI Bakker A Schrieken BAL , et al. Screening for trauma-related symptoms via a smartphone app: the validity of smart assessment on your mobile in referred police officers. Int J Methods Psychiatr Res 2017; 26: e1579. 10.1002/mpr.1579 PMC5639363 28948699

[35] Wang K Varma DS Prosperi M . A systematic review of the effectiveness of mobile apps for monitoring and management of mental health symptoms or disorders. J Psychiatr Res 2018; 107: 73–78. 30347316 10.1016/j.jpsychires.2018.10.006

[36] Henson P Wisniewski H Hollis C , et al. Digital mental health apps and the therapeutic alliance: initial review. BJPsych Open 2019; 5: e15. 10.1192/bjo.2018.86 PMC6381418 30762511

[37] Weisel KK Fuhrmann LM Berking M , et al. Standalone smartphone apps for mental health—a systematic review and meta-analysis. npj Digit Med 2019; 2: 118. 31815193 10.1038/s41746-019-0188-8 PMC6889400

[38] O’Toole K Brown CA . Evaluating the quality of resilience apps for military members and public safety personnel. Journal of Military, Veteran and Family Health 2021; 7: 87–101.

[39] Voth M Chisholm S Sollid H , et al. Efficacy, effectiveness, and quality of resilience-building mobile health apps for military, veteran, and public safety personnel populations: scoping literature review and app evaluation. JMIR mHealth uHealth 2022; 10: e26453. 10.2196/26453 PMC8811698 35044307

[40] Edbrooke-Childs J Edridge C Averill P , et al. A feasibility trial of power up: smartphone app to support patient activation and shared decision making for mental health in young people. JMIR mHealth uHealth 2019; 7: e11677. 10.2196/11677 PMC6682268 31165709

[41] MacLeod L Suruliraj B Gall D , et al. A mobile sensing app to monitor youth mental health: observational pilot study. JMIR mHealth uHealth 2021; 9: e20638. 10.2196/20638 PMC8579216 34698650

[42] Schueller SM Wasil AR Bunyi J , et al. Mental health apps for children and adolescents: a clinician-friendly review. J Am Acad Child Adolesc Psychiatry 2024; 63: 389–392.e1. 38123125 10.1016/j.jaac.2023.07.1004

[43] Shorey S Law E Mathews J , et al. Evaluating the effectiveness of the supportive parenting app on parental outcomes: randomized controlled trial. J Med Internet Res 2023; 25: e41859. 10.2196/41859 PMC9887516 36645699

[44] Hicks LJ Toombs E Lund J , et al. Expanding our understanding of digital mental health interventions for Indigenous youth: an updated systematic review. J Telemed Telecare 2024; 31: 1357633X241239715. 10.1177/1357633X241239715 PMC12301528 38584397

[45] Kim K Au-Yeung A Dagher D , et al. Exploring the relevance of a psychology-based resilience app (JoyPop^TM^) for Indigenous youth. Child Abuse Negl 2024; 148: 106343. 37451896 10.1016/j.chiabu.2023.106343

[46] Povey J Mills PPJR Dingwall KM , et al. Acceptability of mental health apps for aboriginal and Torres Strait Islander Australians: a qualitative study. J Med Internet Res 2016; 18: e65. 10.2196/jmir.5314 PMC4825593 26969043

[47] Chow PI Showalter SL Gerber M , et al. Use of mental health apps by patients with breast cancer in the United States: pilot Pre-post study. JMIR Cancer 2020; 6: e16476. 10.2196/16476 PMC7191345 32293570

[48] Lau N Palermo TM Zhou C , et al. Mobile app promoting resilience in stress management for adolescents and young adults with cancer: protocol for a pilot randomized controlled trial. JMIR Res Protoc 2024; 13: e57950. 10.2196/57950 PMC11322713 39079108

[49] Rincon E Monteiro-Guerra F Rivera-Romero O , et al. Mobile phone apps for quality of life and well-being assessment in breast and prostate cancer patients: systematic review. JMIR mHealth uHealth 2017; 5: e187. 10.2196/mhealth.8741 PMC5735250 29203459

[50] Lai L Sanatkar S Mackinnon A , et al. Testing the effectiveness of a mobile smartphone app designed to improve the mental health of junior physicians: protocol for a randomized controlled trial. JMIR Res Protoc 2024; 13: e58288. 10.2196/58288 PMC11450347 39298756

[51] Sanatkar S Counson I Mackinnon A , et al. Preliminary investigation of shift, a novel smartphone app to support junior doctors’ mental health and well-being: examination of symptom progression, usability, and acceptability after 1 month of use*.* J Med Internet Res 2022; 24: e38497. 10.2196/38497 PMC9536518 36129745

[52] Deady M Glozier N Collins D , et al. The utility of a mental health app in apprentice workers: a pilot study. Front Public Health 2020; 8: 389. 33014953 10.3389/fpubh.2020.00389 PMC7498639

[53] Vilendrer S Amano A Brown Johnson CG , et al. An app-based intervention to support first responders and essential workers during the COVID-19 pandemic: needs assessment and mixed methods implementation study. J Med Internet Res 2021; 23: e26573. 10.2196/26573 PMC8139393 33878023

[54] Bush NE Ouellette G Kinn J . Utility of the T2 mood tracker mobile application among Army Warrior Transition Unit service members. Mil Med 2014; 179: 1453–1457. 25469967 10.7205/MILMED-D-14-00271

[55] Bush NE Dobscha SK Crumpton R , et al. A virtual hope box smartphone app as an accessory to therapy: proof-of-concept in a clinical sample of veterans. Suicide Life Threat Behav 2015; 45: 1–9. 24828126 10.1111/sltb.12103

[56] Bush NE Smolenski DJ Denneson LM , et al. A virtual hope box: randomized controlled trial of a smartphone app for emotional regulation and coping with distress. PS (Wash DC) 2017; 68: 330–336. 10.1176/appi.ps.201600283 27842473

[57] Jaworski BK Ramsey KM Taylor K , et al. Mental health apps and U.S. Military veterans: perceived importance and utilization of the National Center for Posttraumatic Stress Disorder app portfolio. Psychol Serv 2024; 21: 538–551. 37917475 10.1037/ser0000806

[58] Linden B Tam-Seto L Stuart H . Adherence of the #Here4U app – military version to criteria for the development of rigorous mental health apps. JMIR Form Res 2020; 4: e18890. 10.2196/18890 PMC7330732 32554374

[59] Parkes S Croak B Brooks SK , et al. Evaluating a smartphone app (MeT4VeT) to support the mental health of UK armed forces veterans: feasibility randomized controlled trial. JMIR Ment Health 2023; 10: e46508. 10.2196/46508 PMC10495851 37639295

[60] Foley G Ricciardelli R . Views on the functionality and use of the PeerConnect app among public safety personnel: qualitative analysis. JMIR Form Res 2023; 7: e46968. 10.2196/46968 PMC10660208 37930765

[61] Ménard F Ouellet-Morin I Guay S . Effectiveness of mobile apps and text messaging as an E-mental health intervention for the three most common operational stress injuries among public safety personnel: a review of meta-analyses and systematic reviews. Sante Ment Que 2021; 46: 251–275. 34597497

[62] Price JAB Landry CA Sych J , et al. Assessing the perceptions and impact of critical incident stress management peer support among firefighters and paramedics in Canada. IJERPH 2022; 19: 4976. 35564374 10.3390/ijerph19094976 PMC9100761

[63] Stelnicki AM Jamshidi L Fletcher AJ , et al. Evaluation of before operational stress: a program to support mental health and proactive psychological protection in public safety personnel. Front Psychol 2021; 12: 511755. 34484013 10.3389/fpsyg.2021.511755 PMC8416101

[64] Horan KA Marks M Ruiz J , et al. Here for my peer: the future of first responder mental health. IJERPH 2021; 18: 11097. 34769617 10.3390/ijerph182111097 PMC8582745

[65] Jones C Smith-MacDonald L Pike A , et al. Workplace reintegration facilitator training program for mental health literacy and workplace attitudes of public safety personnel: pre-post pilot cohort study. JMIR Form Res 2022; 6: e34394. 10.2196/34394 PMC9092236 35471413

[66] Milliard B . Utilization and impact of peer-support programs on police officers’ mental health. Front Psychol 2020; 11: 1686. 32765375 10.3389/fpsyg.2020.01686 PMC7381167

[67] Anderson GS Di Nota PM Groll D , et al. Peer support and crisis-focused psychological interventions designed to mitigate post-traumatic stress injuries among public safety and frontline healthcare personnel: a systematic review. IJERPH 2020; 17: 7645. 33092146 10.3390/ijerph17207645 PMC7589693

[68] Price JAB Ogunade AO Fletcher AJ , et al. Peer support for public safety personnel in Canada: towards a typology. IJERPH 2022; 19: 5013. 35564405 10.3390/ijerph19095013 PMC9104081

[69] Lazard AJ Saffer AJ Horrell L , et al. Peer-to-peer connections: perceptions of a social support app designed for young adults with cancer. Psycho-Oncology 2020; 29: 173–181. 31483913 10.1002/pon.5220

[70] Aschbrenner KA Naslund JA Shevenell M , et al. A pilot study of a peer-group lifestyle intervention enhanced with mHealth technology and social media for adults with serious mental illness. J Nerv Ment Dis 2016; 204: 483–486. 27233056 10.1097/NMD.0000000000000530 PMC4887192

[71] Aschbrenner KA Naslund JA Shevenell M , et al. Feasibility of behavioral weight loss treatment enhanced with peer support and mobile health technology for individuals with serious mental illness. Psychiatr Q 2016; 87: 401–415. 26462674 10.1007/s11126-015-9395-x PMC4929042

[72] Biagianti B Schlosser D Nahum M , et al. Creating live interactions to mitigate barriers (CLIMB): a mobile intervention to improve social functioning in people with chronic psychotic disorders. JMIR Ment Health 2016; 3: e52. 10.2196/mental.6671 PMC5192235 27965190

[73] Fortuna KL DiMilia PR Lohman MC , et al. Feasibility, acceptability, and preliminary effectiveness of a peer-delivered and technology supported self-management intervention for older adults with serious mental illness. Psychiatr Q 2018; 89: 293–305. 28948424 10.1007/s11126-017-9534-7 PMC5874159

[74] Mueller NE Panch T Macias C , et al. Using smartphone apps to promote psychiatric rehabilitation in a peer-led community support program: pilot study. JMIR Ment Health 2018; 5: e10092. 10.2196/10092 PMC6115596 30111526

[75] Kruzan KP Whitlock J Bazarova NN , et al. Use of a mobile peer support app among young people with nonsuicidal self-injury: small-scale randomized controlled trial. JMIR Form Res 2022; 6: e26526. 10.2196/26526 PMC8787664 35006076

[76] Kruzan KP Whitlock J Chapman J , et al. Young adults’ perceptions of 2 publicly available digital resources for self-injury: qualitative study of a peer support app and web-based factsheets. JMIR Form Res 2023; 7: e41546. 10.2196/41546 PMC9880808 36633896

[77] Goraya NK Alvarez E Young M , et al. Peeroncall: exploring how organizational culture shapes implementation of a peer support app for public safety personnel. Compr Psychiatry 2024; 135: 152524. 39146608 10.1016/j.comppsych.2024.152524

[78] Mickles MS . Helping our heroes: an evaluation of mental health and organizational policies surrounding suicide prevention and postvention strategies for Nebraska first responders. University of Nebraska Medical Center, 2022.

[79] McKeon G Steel Z Wells R , et al. A mental health-informed physical activity intervention for first responders and their partners delivered using Facebook: mixed methods pilot study. JMIR Form Res 2021; 5: e23432. 10.2196/23432 PMC8103303 33885376

[80] Carroll JK Moorhead A Bond R , et al. Who uses mobile phone health apps and does use matter? A secondary data analytics approach. J Med Internet Res 2017; 19: e125. 10.2196/jmir.5604 PMC5415654 28428170

[81] Krakauer RL Stelnicki AM Carleton RN . Examining mental health knowledge, stigma, and service use intentions among public safety personnel. Front Psychol 2020; 11: 949. 32547443 10.3389/fpsyg.2020.00949 PMC7273931

[82] Paradis S Roussel J Bosson J-L , et al. Use of smartphone health apps among patients aged 18 to 69 years in primary care: population-based cross-sectional survey. JMIR Form Res 2022; 6: e34882. 10.2196/34882 PMC9247815 35708744

[83] Ricciardelli R Johnston MS Mario B . The moral impacts of organizational stress on correctional officers. Crim Justice Rev 2024.

