# Supplementary material for: Views on the usability and usefulness of the PeerConnect app among Ontario public safety professionals
Source: Digit Health. 2025 Oct 1;11:20552076251384142. doi: 10.1177/20552076251384142 (PMC12489217; doi:10.1177/20552076251384142)
Supplement: sj-docx-1-dhj-10.1177_20552076251384142 - Supplemental material for Views on the usability and usefulness of the PeerConnect app among Ontario public safety professionals [file sj-docx-1-dhj-10.1177_20552076251384142.docx]

# **Survey Questions**

**Consent**

1. Do you want to participate in our study?

**Employer**

1. In which of the following agencies do you currently work at?
2. If you work at more than one organization, please select your primary organization.

**General Use**

1. Do you use the app PeerConnect?
2. When did you start using the app PeerConnect?
3. How often do you use the app PeerConnect?
4. What feature of the PeerConnect app do you use the most?
5. Do you have any privacy concerns when using the app PeerConnect?
6. Did you find the app PeerConnect easy to download?
7. Do you find the app PeerConnect easy to use?
8. How useful is the app PeerConnect in serving your well-being?
9. How satisfied are you with PeerConnect?

**Newsfeed**

1. How often do you check the NEWSFEED feature?
2. How useful is the information transmitted via NEWSFEED in serving your well-being?
3. How satisfied are you with the NEWSFEED feature?

**Resources**

1. How often do you use the RESOURCES feature?
2. Have you ever used any of the resources listed in the RESOURCES feature?
3. How useful is the RESOURCES feature in serving your well-being?
4. How satisfied are you with the RESOURCES feature?

**Events**

1. How often do you check the EVENTS feature?
2. Have you ever attended any of the events advertised through the EVENTS feature?
3. How useful is the EVENTS feature in serving your well-being?
4. How satisfied are you with the EVENTS feature?

**Self-Assessment**

1. Have you ever used the SELF-ASSESSMENT tool?
2. How useful is the SELF-ASSESSMENT tool in serving your well-being?
3. How satisfied are you with the SELF-ASSESSMENT tool?

**Connect**

1. Have you ever used the CONNECT feature?
2. What is your primary role using the CONNECT feature?
3. How many times have you sought peer support through the CONNECT feature? Please select a number.
4. How useful is the CONNECT feature in serving your well-being?
5. How satisfied are you with the CONNECT feature?

**Overall Evaluation**

1. I prefer seeking peer support from a trusted co-worker without using an app.*
2. I believe employers use apps like the Peer-Connect app to spy on employees.*
3. I don’t have time to access the internet during my shift.*
4. Health apps remind me to care for my well-being.*
5. I have time to practice self-care during my shift.*
6. I feel supported when someone at work shows they care about my well-being.*
7. I believe my organization genuinely cares about my mental health.*
8. I avoid using health apps because I have internet fatigue.*
9. In your opinion, should your organization continue providing access to the app PeerConnect?

**Non-Users**

1. Have you ever used PeerConnect? (Only participants who answered “no” to question 4)
2. Would you use it again? (Only participants who answered “no” to question 4)
3. Do you use any “health app” to help with your mental health? (Only participants who answered “no” to question 4)
4. Did anyone at your organization invite you to try the app Peer-Connect? (Only participants who answered “no” to question 4)
5. Do you believe an app can support your well-being? (Only participants who answered “no” to question 4)

**Demographics**

1. What is your sex assigned at birth?
2. What is your gender?
3. How old are you?
4. What is your marital status?
5. What is your highest level of education completed?
6. How many hours per week do you usually work at the organization you selected at the beginning of this survey? Please enter a number.
7. How often do you access the internet at work?
8. How often do you work in remote areas with limited internet access?
9. How do you describe your knowledge of online technologies?
10. How would you describe your mental health in the last three months?

**Follow-Up**

1. We would like to hear more details about your opinions of PeerConnect. Would you like to participate in a follow-up interview, which may be in a group or individual format, about the usefulness of PeerConnect (sometime between April and May 2022)? Your participation would be voluntary and confidential. If you accept, we will need your first name and email, so we can contact you. (link included for participants to provide their contact information)

*Participants were asked to indicate whether they strongly agree, agree, were neutral, disagree, and strongly disagree with the statement.
